# Microdeletion of 6q16.1 encompassing EPHA7 in a child with mild neurological abnormalities and dysmorphic features: case report

**DOI:** 10.1186/1755-8166-2-17

**Published:** 2009-08-07

**Authors:** Ryan N Traylor, Zheng Fan, Beth Hudson, Jill A Rosenfeld, Lisa G Shaffer, Beth S Torchia, Blake C Ballif

**Affiliations:** 1 Signature Genomic Laboratories, Spokane, WA, USA; 2 Department of Pediatrics, Division of Genetics & Metabolism, University of North Carolina-Chapel Hill, Chapel Hill, NC, USA; 3 Department of Neurology, University of North Carolina-Chapel Hill, Chapel Hill, NC, USA

## Abstract

**Background:**

Of the fewer than 100 cases reported within the literature of constitutional deletions involving the long arm of chromosome 6, only five have been characterized using high-resolution microarray analysis. Reported 6q deletion patients show a high incidence of mental retardation, ear anomalies, hypotonia, and postnatal growth retardation.

**Results:**

We report a 16-month-old male presenting with developmental delay and dysmorphic features who was found by array-based comparative genomic hybridization (aCGH) to have a ~2.16 Mb *de novo *deletion within chromosome band 6q16.1 that encompasses only two genes. Expression studies of the mouse homologue of one of the genes, the ephrin receptor 7 gene (*EPHA7*), have shown the gene functions during murine embryogenesis to form cortical domains, determine brain size and shape, and play a role in development of the central nervous system (CNS).

**Discussion:**

Our results suggest that deletion of *EPHA7 *plays a role in the neurologic and dysmorphic features, including developmental delay, hypotonia, and ear malformations, observed in some 6q deletion patients.

## Background

Conventional cytogenetic analyses have identified fewer than 100 individuals with constitutional deletions within 6q. A review by Hopkin et al. [1] of 57 previously reported 6q deletion cases characterized cytogenetically attempted to organize phenotype/karyotype correlations into three phenotypic groups. Deletions within 6q11 to 6q16, designated Group A, showed a high incidence of hernias, upslanting palpebral fissures, and thin lips with a lower frequency of microcephaly, micrognathia, and heart malformations. Group B deletions spanned 6q15-6q25 and showed increased intrauterine growth retardation, abnormal respiration, hypertelorism, and upper limb malformations. Group C comprised deletions in 6q25 to 6qter, which presented with retinal abnormalities, cleft palate, and genital hypoplasia. Mental retardation was the only finding common among all cases of 6q deletion. The three groups also shared ear anomalies, hypotonia, and postnatal growth retardation in 90%, 82%, and 68% of cases, respectively [1].

Since the Hopkin et al. [1] review, 10 individuals with deletions encompassing 6q16.1 identified using aCGH have been reported [2-7]. Features seen among these cases are varied as the sizes of these deletions span 6–34 Mb within 6q. Here, we report an individual with a ~2.1 Mb deletion within 6q16.1 characterized by high-resolution oligonucleotide microarray analysis. Within this deletion is only one known OMIM gene, the ephrin receptor 7 gene, *EPHA7*. A comparison of the clinical features in the individual reported here to those of previously reported individuals suggests that deletion of *EPHA7 *may play a role in neurodevelopmental deficits in individuals with deletions of 6q16.1.

## Case presentation

The proband presented at 15 months for genetic evaluation of microcephaly and developmental delay. He was born to a 21-year-old mother and 22-year-old father at 38 weeks' gestation with birth weight at the 3^rd ^percentile and length at the 50^th ^percentile. Choroid plexus cysts were detected prenatally by ultrasound but pregnancy and labor were uneventful.

At 6–7 months of age, the proband was not sitting and was noted to have a mild generalized hypotonia. He had received physical therapy since age 6 months and showed mild developmental delay in gross motor skills and multiple other areas. The child was physically small with microcephaly and short stature. At age 10 months, brain MRI showed questionable delayed myelination but upon a second opinion was found to be normal.

Physical examination at 12 months of age showed height at 70.4 cm (2^nd ^percentile), weight at 7.6 kg (<3^rd ^percentile), head circumference 43.3 cm (1^st ^percentile).

**Figure 1:**
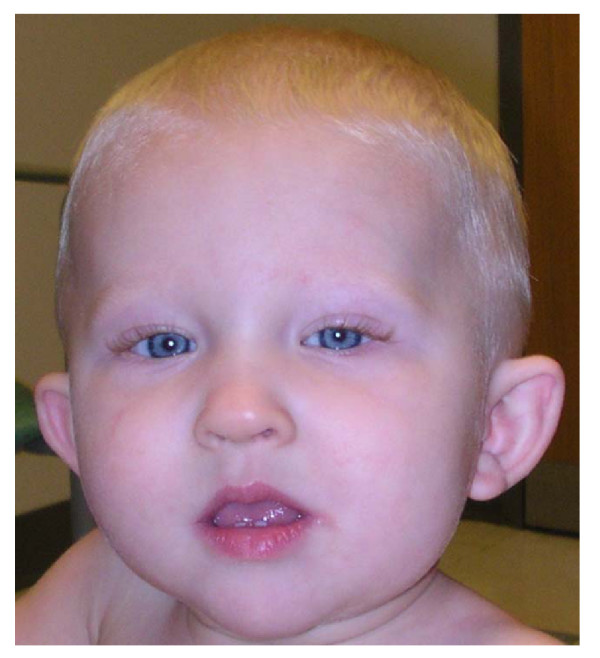
**Proband at age 15 months**. Note triangular-shaped face, mild ptosis on the left side, and cupped, posteriorly rotated ears.

At 15 months of age physical examination showed his height to be at 74.1 cm (3^rd ^percentile; -1.8 SD), weight at 8.06 kg (<3^rd ^percentile; -3.1 SD), and head circumference at 44 cm (<3^rd ^percentile; -2.6 SD). In addition to the short stature, he had generalized hypotonia, mild ptosis, periauricular tags, and cupped ears (Figure 1) with an apparently static encephalopathy. Even though he had a low weight he did not look malnourished, and his head shape was normal. It is noted that the mother is about 1.58 m tall with a head circumference within the 5^th^–10^th ^percentile for a normal female adult.

**Figure 2:**
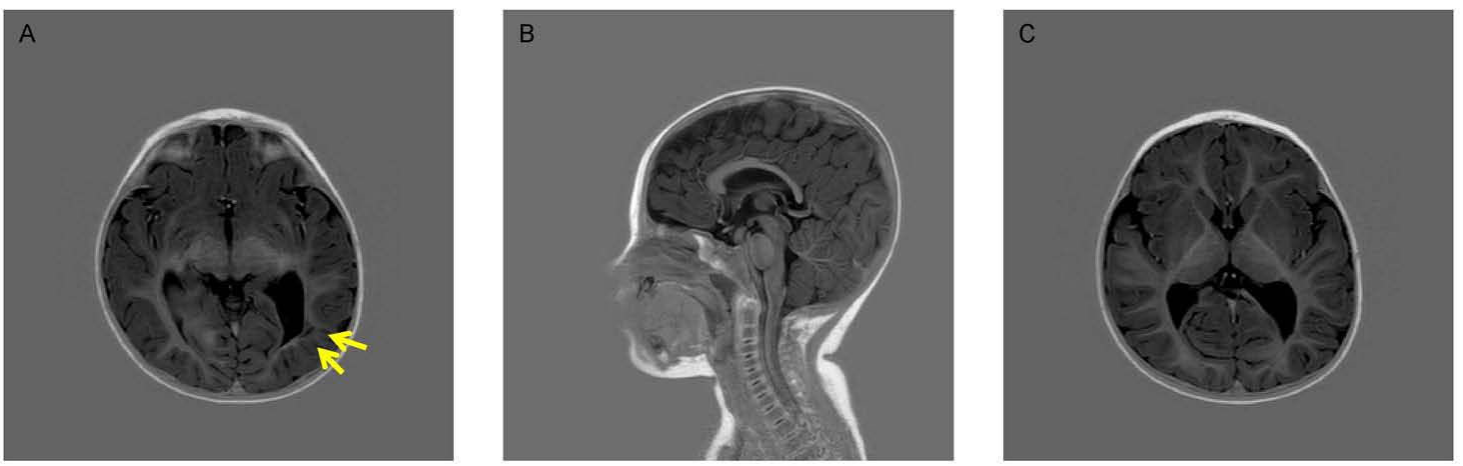
**Brain MRI showing (A) possible brain atrophy and possible hemorrhage, (B) slight thinning of the corpus collosum posteriorly and (C) decrease in white matter volume seen posteriorly in the cerebral hemispheres bilaterally**.

A second brain MRI at 15 months indicated mild generalized brain atrophy and a focal leukomalacia in the periventricular region of the left occipital lobe which likely represents an old hemorrhage (arrows) (Figure 2A). In addition, there was indication of a mild thinning of the corpus callosum posteriorly (Figure 2B) and a bilateral decrease in white matter volume of posterior cerebral hemispheres (Figure 2C).

Karyotype analysis at the 550-band level was normal. Metabolic workup, renal studies, and echocardiogram were essentially normal.

## Microarray results

**Figure 3:**
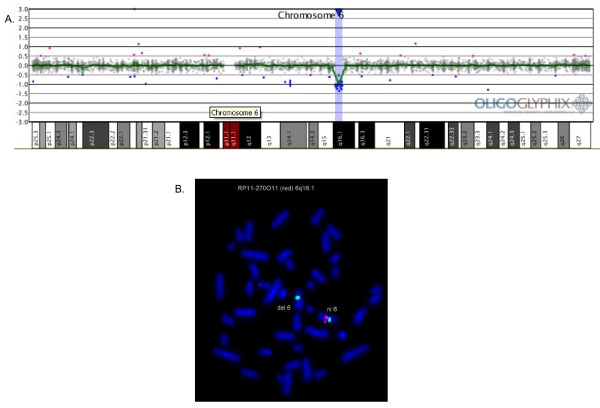
**Analysis of deletion encompassing *EPHA7 *on 6q16.1**. **(A) **Oligonucleotide microarray plot showing a copy-number loss of 30 oligo probes spanning 2.16 Mb at 6q16.1 (chr6:92,836,995–94,905,920). Probes are ordered on the x-axis according to physical mapping positions with distal 6p to the left and distal 6q to the right. **(B) **FISH demonstrating deletion of *EPHA7*. BAC clone RP11-270O11 which encompasses *EPHA7 *was labeled in red and the centromere probe to chromosome 6 (D6Z1) was labeled in green as a control. One red signal was present, indicating deletion of *EPHA7*.

Oligonucleotide aCGH performed on DNA from the proband's peripheral blood identified a copy-number loss of 30 oligo probes spanning ~2.16 Mb at 6q16.1 (chr6:92,836,995–94,905,920). The nearest distal oligonucleotide probe on chromosome 6 that was not deleted was ~83.3 kb away from the deleted region, and the nearest proximal oligonucleotide probe that was not deleted was ~56.4 kb away from the deleted region; there are no genes within either gap (Figure 3A). Fluorescence *in situ *hybridization (FISH) using BAC clone RP11-270O11 which encompasses *EPHA7 *confirmed the deletion (Figure 3B). FlSH analysis was performed on both parents and neither parent was found to carry a deletion or translocation of the 6q16.1 region; thus, the proband's deletion is apparently *de novo *in origin.

## Discussion

We report a 15-month-old male with a ~2.16 Mb interstitial deletion of 6q16.1 characterized by high-resolution oligonucleotide microarray. Clinical features include developmental delay, microcephaly, short stature, congenital hypotonia, mild ptosis, periauricular tags, cupped ears, and abnormal brain MRI. A comparison of the clinical features observed in our patient and the previously reported cases identified by aCGH that clearly overlap the current case shows several common features including developmental delay (4/4), microcephaly (2/4), malformed ears (4/4), microretrognathia (3/4), and hypotonia (4/4) (Table 1). Hopkin et al. [1] found that the typical facial features of patients with deletion breakpoints within 6q15-6q25 (Group B) included prominent forehead, microcephaly, microretrognathia, short palpebral fissures, and prominent nose.

**Table 1:** Summary of clinical features of individuals with 6q16.1 deletions encompassing *EPHA7 *characterized by aCGH

	Klein et al[2] Patient 3	Le Caignec et al[4]	Zherebstov et al[3]	Current Case
Sex	Male	Female	Female	Male
**Array Results**				
Array type	BAC	BAC	Oligo	Oligo
Deletion size	11.3–15.7 Mb	12.9–15.7 Mb	34 Mb	2.1 Mb
Breakpoints	q15-q21	q15-q21	q16.1-q22.23	q16.1-q16.1
**Birth Measurements**				
Gestational age	Not Reported	40 weeks	39 weeks	38 weeks
Weight	90%	5%	-3.8 SD	3^rd ^percentile
Length	90%	25–50%	50%	50^th ^percentile
HC	NA	NA	-3 SD	NA
				
**Current Growth Parameters**				
Age	13 years	32 months	7 months	15 months
Weight	NA	50%	10th percentile	-3.1 SD
Height	NA	25–50%	-2 SD	-1.8 SD
HC	>95^th ^percentile	50^th ^percentile	10th percentile	<3^rd ^percentile
				
**Neurological Features**				
Head MRI results	Normal	Normal	CT: subdural hemorrhage due to significant brain atrophy	Questionable brain atrophy/Questionable hemorrhage
Ptosis	NA	NA	NA	Mild on left
Eye/Vision anomalies	Myopia	Strabismus/hypermetropia	Poor visual motor development	NA
Developmental delay	Present	Present	Present	Present
Microcephaly	Absent	Absent	Present (-3 SD)	Present (-2 SD)
Head control			Head tremor	
Face shape	Bitemporal narrowing	Not reported	Flat occiput/heart-shaped face	Triangular-shaped face
				
**Dysmorphic Features**				
Interpupillary distance	Hyperteloric	Hyperteloric	Hyperteloric	Normal
Epicanthal folds	Present	NA	Absent	Absent
Downslanting palpebral fissures	Absent	Absent	Present	Absent
Malformed ears	Low-set	Upturned, angulated	Low-set/posteriorly rotated with hypoplastic helices	Cupped ears, periauricular tags
Microretrognathia	Present	Present	Present	Absent
				
**Organ Malformation**				
Renal/genitourinary anomalies	Post-urethral valve	NA	Bilateral echogenic kidneys/pelvicaliectasis of the right kidney	Normal
Cardiac anomalies	Normal	NA	Tetrology of Fallot	Normal
Hypotonia	Present	Present	Present	Present

HC: head circumference; NA: not available; SD: standard deviation

The deletion identified in our case encompasses two genes. One gene, *TSG1*, a predicted tumor suppressor gene [8], is not a likely candidate for the clinical features in our patient. The remaining gene, ephrin receptor 7 (*EPHA7*) (chr6:94,006,458–94,186,021, UCSC build March 2006 – hg18) is part of the Eph/ephrin family of receptor tyrosine kinases (RTK), cell-surface-bound proteins whose signaling mediates cell-to-cell communication during development and directs the migration and positioning of cells within many tissue types [9]. The complexity of Eph/ephrin signaling is demonstrated by the proteins' involvement in bidirectional signaling, participation in a broad spectrum of developmental processes, and as a player in other communicative pathways [10]. Murine studies have shown *EphA7 *functions during embryogenesis to form cortical domains, determine brain size and shape, and play a role in development of the CNS [8-10].

**Figure 4:**
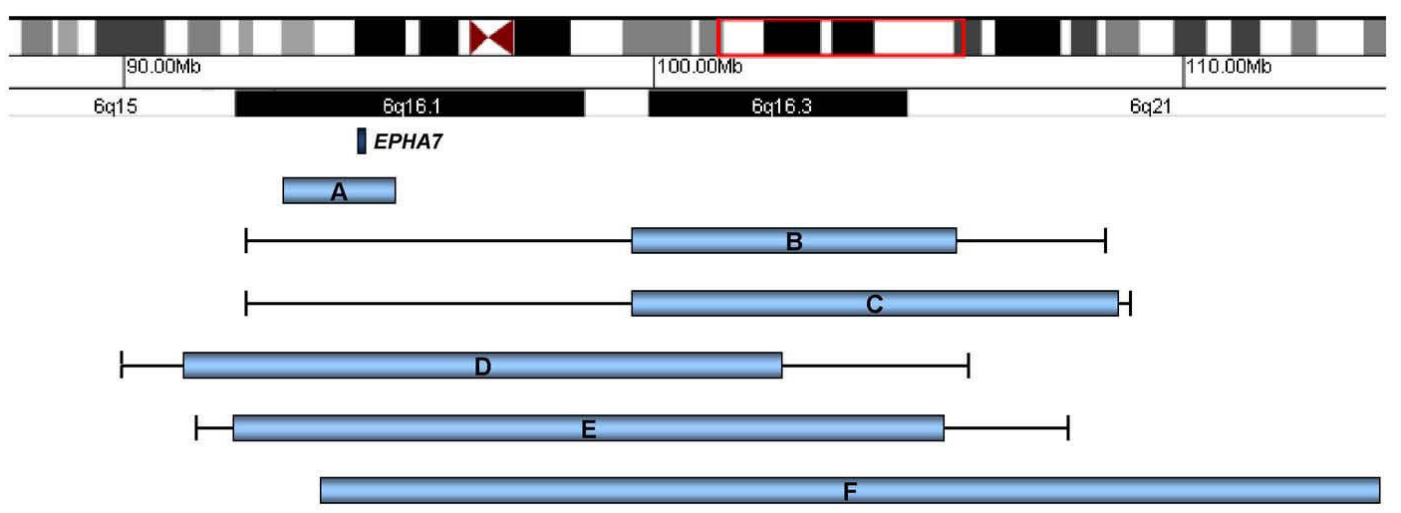
**Zoomed view of 6q15-6q21**. Blue bars represent minimal deletion sizes; black lines represent maximum deletion sizes for each patient based on gaps in microarray coverage. Bar A is the current case with a deletion size of 2.1 Mb. Bar B is Klein et al. [2] patient 2 with a deletion size of 6–16.2 Mb; Bar C is Klein et al. [2] patient 1 with deletion of size 8.8–16.4 Mb; Bar D is Klein et al. [2] patient 3 with deletion size of 11.3–15.7 Mb; Bar E is the patient presented by Le Caignec et al. [4] with deletion size of 12.9–15.7 Mb; Bar F is the patient presented by Zherebstov et al. [3] with deletion size of 34 Mb (the bar image for this deletion extends beyond the coordinates depicted). The dark blue band represents *EPHA7*.

Of the five previously reported 6q deletions detected by aCGH, three are presumed to encompass *EPHA7*. Patient 3 reported by Klein et al. [2] and individuals reported by Le Caignec et al. [4] and Zherebstov et al. [3] had deletions identified by aCGH with clones covering the 6q16.1 region (Figure 4). Common features among these cases and the current case include developmental delay, ear anomalies, and hypotonia (Table 1). Patients 1 and 2 reported by Klein et al. [2] had deletions ranging from 6–16.4 Mb. However, it is not known whether *EPHA7 *is deleted in these two cases because the gene lies between the most proximal BAC probe known to be deleted and the next-nearest probe, which is retained, whereas the deletion observed in Klein et al. [2] patient 3 had a proximal breakpoint that lies within band q15 (Figure 4) and thus encompasses *EPHA7*. In addition, the deletions seen in the other cases characterized by aCGH are much larger and thus include many more genes. Although the exact breakpoints of these previously reported large deletions cases are unknown, the deletion of only one known gene in our patient and the presence of clinical features, including hypotonia, abnormal or low-set ears, and developmental delay, that are similar to those of the individuals reported by Klein et al. [2], suggest *EPHA7 *plays a role in central nervous system function.

This current case is the smallest deletion encompassing 6q16 reported, to the best of our knowledge, within the literature to date. Phenotypic overlap between previously reported 6q16 deletion cases that have been characterized at the molecular level by array CGH with this case indicates that in humans *EPHA7 *plays a role in neurological and dysmorphic features such as developmental delay, hypotonia, and ear malformations. The characterization of additional cases at the molecular level will assist with accurate genotype/phenotype correlations and allow for the identification of additional features associated with 6q16 deletions.

## Materials and methods

### Oligonucleotide aCGH

Oligonucleotide-based microarray analysis was performed using a 105K-feature whole-genome microarray (SignatureChip Oligo Solution^®^, made for Signature Genomic Laboratories by Agilent Technologies) with one probe every 10 kb in targeted regions – microdeletion/microduplication syndromes, the pericentromeric regions, subtelomeres and genes in important developmental pathways – and an average probe spacing of one probe every 35 kb throughout the rest of the genome. Two oligonucleotide probes cover the *EPHA7 *gene which spans ~178 kb. Genomic DNA was labeled with Alexa Fluor dyes 555 or 647 using a BioPrime Total DNA labeling kit (Invitrogen Corp, Carlsbad, CA). Array hybridization and washing were performed as specified by the manufacturer (Agilent Technologies). Arrays were scanned using an Axon 4000B scanner (Molecular Devices, Sunnyvale, CA) and analyzed using Agilent Feature Extraction software v9.5.1 and Agilent CGH Analytics software v3.5.14. Results were then displayed using custom oligonucleotide aCGH analysis software (Oligoglyphix™; Signature Genomic Laboratories).

### FISH Analysis

The deletion was confirmed and visualized by metaphase fluorescence *in situ *hybridization (FISH) using a bacterial artificial chromosome (BAC), clone RP11-270O11, which encompasses the *EPHA7 *locus. Metaphase chromosomes were obtained from the proband's sample by using standard culturing procedures, and fixed cell suspensions were dropped onto clean microscope slides. After a 10 min 2× SSC soak the slides were dehydrated in a series of ethanol washes (70%, 90%, and 95%) for 2 min each wash. The slides were then air-dried. The chromosomes were denatured in a 70% formamide, 2 × SSC solution at 70°C for 2 min, immediately placed in 70% ethanol at -20°C for 2 min, then transferred to 80%, 90%, and 100% ethanol at -20°C for 2 min each, and then air-dried. DNA was labeled by nick translation (Abbott Molecular Inc., Des Plaines, IL, USA) with Enzo Red-dUTP or Enzo Green-dUTP (Abbott). Each was diluted in a 50% formamide hybridization solution. The double stranded DNA probes were denatured at 70°C for 10 min and were applied to the prepared slides. Slides were placed in a moist chamber and incubated overnight at 37°C. The slides were washed at 43°C in 0.4× SSC/0.3% Tween solution for 2 min, placed in 2× SSC/0.1% Tween for 1 min. The slides were counterstained with DAPI. Cells were examined with a Zeiss Axioplan II, Imager.M1, or Imager.Z1 fluorescence microscope equipped with a triple-bandpass filter that allows multiple colors to be visualized simultaneously. Digital images were captured and stored with Isis software V 3.4.0 (Metasystems, Altlussheim, Germany).

## Competing interests

Z Fan and B Hudson have no competing interests to declare. RN Traylor, JA Rosenfeld, BS Torchia and BC Ballif are employees of Signature Genomic Laboratories, LLC. LG Shaffer sits on the Members' Board of Signature Genomic Laboratories, LLC, is President & CEO, and owns shares in the company.

## Authors' contributions

RNT wrote the manuscript; BH and ZF referred the patient for study; JAR coordinated analysis of clinical features; BST signed out the molecular cytogenetic results; LGS and BCB coordinated the study. All authors have read and approved the manuscript.

## Consent

This case report is presented with the consent of the patient's family.
